# TLR-9 Plays a Role in *Mycobacterium leprae*-Induced Innate Immune Activation of A549 Alveolar Epithelial Cells

**DOI:** 10.3389/fimmu.2021.657449

**Published:** 2021-08-12

**Authors:** André Alves Dias, Carlos Adriano de Matos e Silva, Camila Oliveira da Silva, Natasha Ribeiro Cardoso Linhares, João Pedro Sousa Santos, Aislan de Carvalho Vivarini, Maria Ângela de Mello Marques, Patrícia Sammarco Rosa, Ulisses Gazos Lopes, Márcia Berrêdo-Pinho, Maria Cristina Vidal Pessolani

**Affiliations:** ^1^ Laboratory of Cellular Microbiology, Oswaldo Cruz Institute, Oswaldo Cruz Foundation (FIOCRUZ), Rio de Janeiro, Brazil; ^2^Laboratory of Molecular Parasitology, Institute of Biophysics Carlos Chagas Filho, Federal University of Rio de Janeiro (UFRJ), Rio de Janeiro, Brazil; ^3^Mycobacteria Research Laboratories, Department of Microbiology, Immunology and Pathology, Colorado State University (CSU), Fort Collins, CO, United States; ^4^Division of Research and Teaching, Lauro de Souza Lima Institute, Bauru, Brazil

**Keywords:** *Mycobacterium leprae*, Toll-like receptor 9 (TLR-9), extracellular DNA, histone-like protein (Hlp), respiratory epithelial cells, innate immune response, chemokines

## Abstract

The respiratory tract is considered the main port of entry of *Mycobacterium leprae*, the causative agent of leprosy. However, the great majority of individuals exposed to the leprosy bacillus will never manifest the disease due to their capacity to develop protective immunity. Besides acting as a physical barrier, airway epithelium cells are recognized as key players by initiating a local innate immune response that orchestrates subsequent adaptive immunity to control airborne infections. However, to date, studies exploring the interaction of *M. leprae* with the respiratory epithelium have been scarce. In this work, the capacity of *M. leprae* to immune activate human alveolar epithelial cells was investigated, demonstrating that *M. leprae*-infected A549 cells secrete significantly increased IL-8 that is dependent on NF-κB activation. *M. leprae* was also able to induce IL-8 production in human primary nasal epithelial cells. *M. leprae*-treated A549 cells also showed higher expression levels of human β-defensin-2 (hβD-2), MCP-1, MHC-II and the co-stimulatory molecule CD80. Furthermore, the TLR-9 antagonist inhibited both the secretion of IL-8 and NF-κB activation in response to *M. leprae*, indicating that bacterial DNA sensing by this Toll-like receptor constitutes an important innate immune pathway activated by the pathogen. Finally, evidence is presented suggesting that extracellular DNA molecules anchored to Hlp, a histone-like protein present on the *M. leprae* surface, constitute major TLR-9 ligands triggering this pathway. The ability of *M. leprae* to immune activate respiratory epithelial cells herein demonstrated may represent a very early event during infection that could possibly be essential to the generation of a protective response.

## Introduction

Leprosy is a chronic infectious disease caused by the obligate intracellular pathogen *Mycobacterium leprae*. The ability of this mycobacterium to infect Schwann cells may result in peripheral nerve damage with severe physical deformities, a hallmark of the disease (1). Despite the overall effectiveness of multidrug therapy (MDT), leprosy remains a public health problem with approximately 200,000 annual new registered cases worldwide (2). Even with the easy availability of MDT, it has largely failed to interrupt disease transmission. Furthermore, attempts to prevent the spread of *M. leprae* are severely constrained by the lack of an effective vaccine with potential prophylactic and therapeutic usages.

The disease is transmitted from person to person and the respiratory tract has been considered the most probable route of bacterial entry (3). It is well established, for example, that, in urban areas, the nasal discharge from leprosy patients with a high bacillary load (multibacillary [MB] patients) is the main source of infection (4). However, most *M. leprae-*infected individuals will generate an effective T helper 1 (Th1)-based immune response upon producing IFN-γ and will never develop any clinical sign of the disease (5, 6).

The epithelium of the respiratory tract serves as the first line of defense against invading airway microorganisms. Besides acting as a physical barrier, respiratory epithelium cells express a variety of pattern recognition receptors (PRRs), including Toll-like (TLRs), RIG-1-like, and NOD-like receptors that recognize the pathogen-associated molecular patterns (PAMPs), initiating a local innate immune response while orchestrating a subsequent adaptive immune response to control the infection. PAMPs recognition triggers signaling pathways mediated by Jak-Stat, NF-κB, and IRFs, leading to the secretion of cytokines, chemokines, reactive nitrogen and oxygen species, and anti-microbial peptides, all of which evoke immune cell recruitment along with a critical immune response for the early control of infection (7).

Over the years, many studies in the mycobacterial field have highlighted the prominent role played by the respiratory epithelium during *Mycobacterium tuberculosis* infection. After adhesion and internalization into epithelial cells, *M. tuberculosis* induces cytokine (such as TNF, IL-6 and IL-10) and chemokine (IL-8, IP-10, IL-27, MCP-1 and MIG) production that allows immune cells to migrate to the infection site and subsequent activation (8–10). However, very little is known about the interactions taking place between *M. leprae* and airway epithelial cells and even less about how these interactions may contribute to the infection outcome.

Interestingly, shortly after airborne or intranasal infection of mice with *M. leprae*, acid-fast bacilli are preferentially found in the lungs, and only rarely observed in the nasal mucosa. This demonstrates that the lungs are the main portal of both the entry and dissemination of the leprosy bacillus (11, 12). In a recent study, the capacity of *M. leprae* to infect nasal and alveolar epithelial cells was confirmed in *in vitro* cell culture assays (12). Moreover, the heparin-binding hemagglutinin (HBHA) protein and the histone-like protein (Hlp), previously implicated in the adhesion and internalization of *M. tuberculosis* in airway epithelial cells (13, 14), were also detected on the *M. leprae* surface and shown to promote mycobacterial interaction with these cells (12, 15).

Besides its role as an adhesin, Hlp, also referred to as a laminin-binding protein (LBP) (16), or mycobacterium DNA-binding protein 1 (MDP-1), was shown to elicit a protective immune response in mice against *M. tuberculosis* (17). This protective activity, occurring only when Hlp was administered together with mycobacterial DNA, and not when DNA or Hlp was injected alone, was dependent on TLR-9 recognition (17). Indeed, TLR-9, which recognizes unmethylated deoxycytidylate-phosphate-deoxyguanylate (CpG) motifs in viral and bacterial DNA (18), has been shown to be an important innate immune pathway for the generation of a protective immune response against *M. tuberculosis* (8, 19, 20). More recently, the *M. leprae*-derived DNA-Hlp complex was shown to activate the TLR-9 pathway (21).

In the present study, the capacity of *M. leprae* to immune activate alveolar epithelial cells by analyzing the secretion of cytokines and chemokines and the nuclear translocation of transcriptional factor NF-κB was tested. Moreover, due to the known involvement of TLR-9 in mycobacterial recognition and its expression in respiratory epithelial cells (8), the participation of this pathway in this activation was also examined. Finally, the potential role of bacterial surface-exposed DNA-Hlp complexes as TLR-9 ligands was explored.

It is hoped that the knowledge generated in this study may contribute to designing an effective leprosy vaccine.

## Materials And Methods

### Mycobacterial Use and Growth Conditions

*M. leprae* (Thai-5 strain), purified from the footpad of athymic mice (*nu/nu*), was kindly provided by Dr. Patrícia Sammarco Rosa from the Lauro de Souza Lima Institute (ILSL, Bauru, SP). Bacilli were γ-irradiated for 5 minutes using two linear electron accelerators, each equipped with 18 kW of power and 10 MeV of energy (Acelétron, Rio de Janeiro, RJ). The *Mycobacterium smegmatis* mc^2^ 155 wild type (wt) and the knockout strain for the hlp gene (*Δhlp*) were donated by Dr. Thomas Dick of the University of Singapore. Mycobacterial strains were cultivated in Middlebrook 7H9 medium (Sigma-Aldrich), supplemented with 10% (v/v) ADC (albumin and dextrose) and 0.05% (v/v) Tween 80 at 37°C, under constant agitation, until the end of the exponential growth phase (OD_600nm_). *M. smegmatis Δhlp* was grown in the presence of 20 µg/mL of kanamycin.

### Cell Culture Conditions

The human alveolar epithelial cell line A549 and the murine macrophage RAW 264.7 were purchased from the American Type Culture Collection (ATCC). The cells were maintained, respectively, in DMEM/F12 and RPMI-1640 culture medium containing 2 mM L-Glutamine (LGC Biotechnology, SP, Brazil), and supplemented with 10% of fetal bovine serum (FBS) (Cultilab, SP, Brazil). Cultures were kept at 37°C within a humidified 5% CO_2_ atmosphere. The A549 cells were seeded for 24 hours at 37°C prior to stimulation in 6-well plates (5 x 10^5^ cells for well) for the PCR assays and 24-well plates (7 x 10^4^ cells for well) for the other assays. In the current study, we also used primary nasal epithelial cells obtained from the nasal polyps of patients submitted to polypectomy for nasal clearing (further details see [12]). The procedures described for the use of nasal polyps were approved by the Pedro Ernesto University Hospital, the State University of Rio de Janeiro, and the Oswaldo Cruz Foundation (FIOCRUZ) Ethical Committee located in Rio de Janeiro, RJ, Brazil. All participants provided their written consent.

### Cell Infection and Stimulation

A549 cells were incubated at different time periods at 37°C. For cell infected with live *M. leprae* cultures were incubated at 33°C. The stimuli used were: 1) live *M. leprae*; 2) irradiated-killed *M. leprae*; 3) CpG oligonucleotide (ODN 2395; 1 µM; Invivogen, San Diego, CA, USA); 4) *M. leprae* rHlp protein (0.5 µM); 5) CpG-rHlp complex; 6) LPS from *Escherichia coli* (1 µg/ml; Sigma-Aldrich, San Luis, MO, USA); and 7) *M. smegmatis* wt and *Δhlp* pre-incubated or not with 1 µM of CpG oligonucleotide. Human primary nasal epithelial cells were incubated only with irradiated-killed *M. leprae* for 24 hours at 37°C. For the experiment with RAW 264.7 cells, the stimuli used were: 1) CpG oligonucleotide (0.5 µM); 2) *M. leprae* rHlp protein (0.25 µM); 3) Hlp synthetic peptides (0.25 µM); 4) CpG-rHlp or -peptides complexes; and 5) LPS (100 ng/ml).

The rHlp protein was incubated with CpG oligo, as previously described (21), to obtain the CpG-Hlp complex. The same protocol was adopted to acquire the CpG-peptide complexes. The Hlp synthetic peptides p2, p3, and p10 (30 amino acids each) were donated by Dr. Tom Ottenhoff of the University Medical Center of Leiden (Netherlands). At the end of each incubation period, the cell culture supernatants were stored at -70°C for subsequent mediators quantification by commercial ELISA kits (R&D Systems, Minneapolis, MN, USA).

For the NF-κB translocation inhibitory experiments, pretreatment of the A549 cells was performed with the drugs wedelolactone (Sigma-Aldrich) at 80 µM or Bay11-7082 (Sigma-Aldrich) at 10 µM. Alternatively, the cells were also transfected with 2.5 µg of the expression plasmid pEGFP-C3 (Clontech, Kusatsu, Shiga, Japan) coding for a dominant-negative form of IκBα (DN-IκBα; kindly provided by Dr. Patrick Baeuerle of the Gene Center, Martinsried, Germany) (22) plus 1.25 ml of Lipofectamine-2000 reagent (Invitrogen, Carlsbad, CA, USA) according to the manufacturer’s instructions for 24 hours followed by washing with PBS and infection or stimulation as described above. For TLR-9 blocking assays, cells were pretreated with the synthetic antagonist E6446 (Eisai, Bunkyo-ku, Tokyo, Japan) at 0.2 µM.

Incubation times varied according to the type of experiment, as specified in the *Results* section. Each experiment was done in duplicate.

### Real-Time Quantitative Reverse Transcription PCR (qRT-PCR)

Total RNA of the treated A549 cells was extracted with the Trizol reagent (Life Technologies, Carlsbad, CA, EUA), according to the protocol provided by the manufacturer. The cDNA was obtained from the total RNA using the reverse transcriptase enzyme Superscript III^®^ (Life Technologies) whereas qRT-PCR was performed using the SYBR Green I system (Applied Biosystems, Waltham, MA, USA), accompanied by specific primers for the coding sequences of the human β-defensin-2 (hβD-2) and 60S ribosomal protein L13 (RPL-13) genes in line with recommendations of the manufacturer, followed by incubating the reactions in the ViiA-7^®^ qPCR system (Applied Biosystems). In each sample, the cDNA of the gene of interest (hβD-2) and the constitutive gene used as a normalizer (RPL-13) were amplified. Gene expression analysis was performed *via* the delta-delta CT (ΔΔCT) method. Once the ΔCT of the samples was determined, the cDNA in the experimental condition of the unstimulated cells was chosen as the normalizing sample. Relative gene expression values were obtained by applying the 2-ΔΔCT formula (23).

### Determination of NF-κB Activation

The A549 cells were resuspended in a hypotonic lysis buffer (10 mmol/L HEPES [pH 7.9], 1.5 mmol/L MgCl_2_, 10 nmol/L KCl, 0.05 mmol/L of PMSF, 0.5 mmol/L of dithiotheitirol, and a cocktail of protease inhibitors at 1x [Complete Mini^®^ - Roche, Basel, Switzerland]) for 15 minutes after which 10% Igepal was added to complete the lysis. The homogenates were centrifuged (13,000 x g) for 30 seconds; and the pellet containing the nuclei was resuspended in a nuclear extraction hypertonic buffer (10 mmol/L HEPES [pH 7.9], 0.42 M NaCl, 1.5 mmol/L MgCl_2_, 10 nmol/L KCl, 0.5 mmol/L PMSF, and 1 mmol/L dithiotheitirol and protease inhibitors). After 40 minutes of continuous stirring, the extracts were centrifuged (13,000 x g) for 10 minutes at 4°C, and the proteins containing the nuclear extracts were quantified *via* the BCA technique (Pierce, Groton, CT, USA). The presence of NF-κB in the nuclear extract was determined by immunoblotting or ELISA.

For electrophoresis, in conjunction with the subsequent immunoblotting step, 10 µg of the nuclear protein extract were applied in gel with 15% polyacrylamide and 0.1% SDS. To perform the immunoblotting, the proteins placed in the gel were transferred to a nitrocellulose membrane (Hybond-C Extra - GE Healthcare, Chicago, IL, USA), incubated for 1 hour with anti-p65 monoclonal antibody (NF-κB subunit; Santa Cruz Biotechnology, Dallas, TX, USA) diluted 1:1000, followed by an additional 1 hour incubation period with anti-mouse IgG conjugated to peroxidase (Sigma-Aldrich) diluted 1:30000. Lamin A/C (1:1000; Santa Cruz Biotechnology) was used as a nuclear marker normalizer. The membrane was developed with the chemiluminescent substrate ECL (GE Healthcare) by exposing the membrane to a film that was later analyzed by densitometry through Image-J^®^ software.

For the ELISA assays, 10 µg of nuclear extract protein were used, as stated in the manufacturer’s protocol (NF-κB p65 ELISA kit; e-Bioscience, Santa Clara, CA, USA), a methodology based on the detection of chemiluminescence caused by the binding of the NF-κB present in the nucleus (detection of the p65 subunit) in the consensus oligonucleotidal sequence attached to the plate. As a positive control, the kit includes proteins from purified nuclear extracts taken from treated cells.

### Flow Cytometry

The A549 cells were detached from the plate with 10 mM EDTA, washed with PBS containing 1% FSB, and incubated with monoclonal antibodies to MHC-II conjugated to allophycocyanine (APC) and CD80 conjugated to phycoerythrin (PE), in addition to their respective isotype controls (all from Biolegend, San Diego, CA, USA), at a concentration of 1:10 (v/v) at 4°C in the absence of light for 30 minutes. Finally, the cell suspension was fixed with 1% paraformaldehyde. The cells were analyzed by way of FACS Accuri flow cytometer (BD Bioscience, Franklin Lakes, NJ, USA), and the resulting data, *via* FlowJo V10 software (Tree Star).

### Binding of Recombinant Hlp to DNA

Polystyrene microtiter plates (Corning Inc., Corning, NY, USA) were covered with 10 µg/mL of mycobacterial genomic DNA obtained from the culture mass of the Pasteur strain of *M. bovis* BCG (24), diluted in buffer carbonate/bicarbonate at 0.1 M (pH 9.6), and incubated overnight at 4°C. The wells were then washed with PBS and blocked for 2 hours at room temperature with PBS containing 3% bovine serum albumin (BSA; Sigma-Aldrich). After washing with PBS containing 0.05% Tween 20 (Sigma-Aldrich; PBS/T), different rHlp concentrations were added to the wells and incubated at 37°C for 2 hours. The wells were then incubated with the monoclonal antibody for Hlp (5G9; 1:500) (25) for 1 hour at 37°C, at which time the wells were once again washed with PBS/T, followed by incubation with peroxidase-conjugated anti-mouse IgG (1:1000; Sigma-Aldrich) for an additional period of 50 minutes at 37°C. Peroxidase activity was revealed by using hydrogen peroxide and tetramethylbenzidine (TMB; LGC Biotechnology). The colorimetric reaction was interrupted with 2.5 N sulfuric acid; and the optical density reading at 450nm was determined on a spectrophotometer *via* the SOFTmax^®^PRO 4.0 program (Life Sciences Edition, Molecular Devices Corporation).

### Statistical Analysis

Statistically significant differences among the values were determined using the GraphPad Prism 5 Project program (GraphPad Software Inc.), after applying the unpaired *t* test, the One-way analysis of the variance test (ANOVA) with Bonferroni, or the linear trend post-test, in which a p value of <0.05 was considered significant.

## Results

### *M. leprae* Induces Immune Activation of Alveolar Epithelial Cells

**Figure 1:**
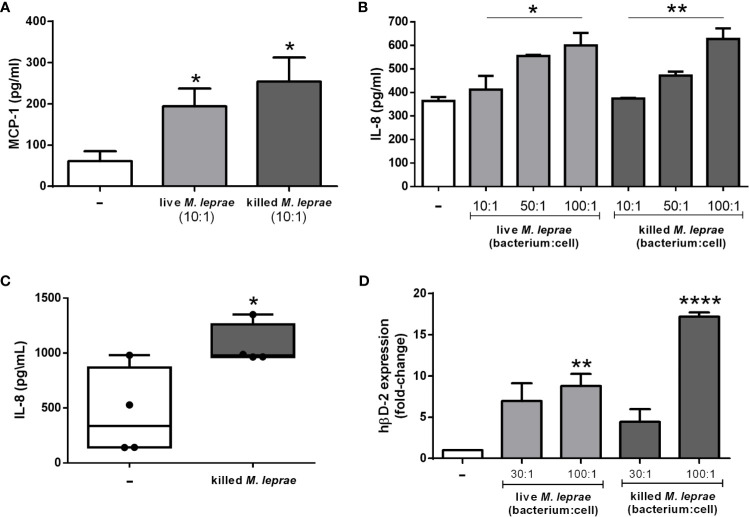
*M. leprae* induces secretion of chemokines and expression of hβD-2 gene in human respiratory epithelial cells. **(A, B)** ELISA quantification of MCP-1 **(A)** and IL-8 **(B)** levels in the culture supernatant of A549 cells stimulated with live or killed *M. leprae* at different bacterium:cell ratios for 24 hours. **(C)** ELISA quantification of IL-8 levels in the culture supernatants of primary human nasal epithelial cells obtained from 4 donors and stimulated with killed *M. leprae* at a bacterium:cell ratio of 10 for 24 hours. **(D)** Quantitative RT-PCR analysis of hβD-2 mRNA levels in A549 cells stimulated with live or killed *M. leprae* at different bacterium:cell ratios for 24 hours. **(A, C, D)** Unpaired *t*-test with differences considered statistically significant in relation to the untreated cells. **(B)** ANOVA test with differences considered statistically significant in relation to the untreated cells comparing the different doses by applying a linear-trend post-test. *p < 0.05; **p < 0.01; ****p < 0.0001. Values ​​represent the mean ± standard deviation of at least 3 independent experiments performed in duplicate.

As a first step, an investigation was launched into whether *M. leprae* was able to induce the secretion of the chemokines MCP-1 (CCL2) and IL-8 (CXCL8) in alveolar type II pneumocytic epithelial A549 cells, known to play an important role in mediating immune cell migration to the infection site (26). For the quantification of MCP-1 and IL-8, the A549 cells were incubated or not with live or irradiated (killed) *M. leprae* for 24 and 48 hours and the chemokines were measured by ELISA in culture supernatants. The results showed that both live and killed *M. leprae* (bacterium:cell ratio of 10) induced the production of MCP-1 in A549 cells in view of the higher MCP-1 levels detected in the supernatants of the treated *versus* control cells after 24 hours of incubation (**Figure 1A**). However, at later time point of incubation (48 hours) only killed bacteria was able to induce MCP-1 (**Supplementary Figure 1A**). Both live and killed *M. leprae* were also able to induce IL-8 in A549 cells, but at higher bacterium:cell ratios (50 and 100), in a dose-dependent manner at 24 and 48 hours (p-values <0.05) (**Figure 1B** and **Supplementary Figure 1B**). To verify whether IL-8 induction also occurs in primary epithelial cells, we incubated human primary nasal epithelial cells isolated from four healthy volunteers with killed *M. leprae for* 24 hours at a bacterium:cell ratio of 10. **Figure 1C** shows that *M. leprae* is also able to induce IL-8 production in primary epithelial cells.

Alveolar epithelial cells are also able to produce such antimicrobial peptides as hβD-2 and LL-37 (8), known to be effective against mycobacteria (27–30). Therefore, as a next step, hβD-2 expression was evaluated by qRT-PCR in *M. leprae*-incubated A549 cells. Cells infected with live or treated with killed *M. leprae* at a bacterium:cell ratio of 100 for 24 hours showed significantly higher expression levels of hβD-2 over unstimulated cells (**Figure 1D**).

**Figure 2:**
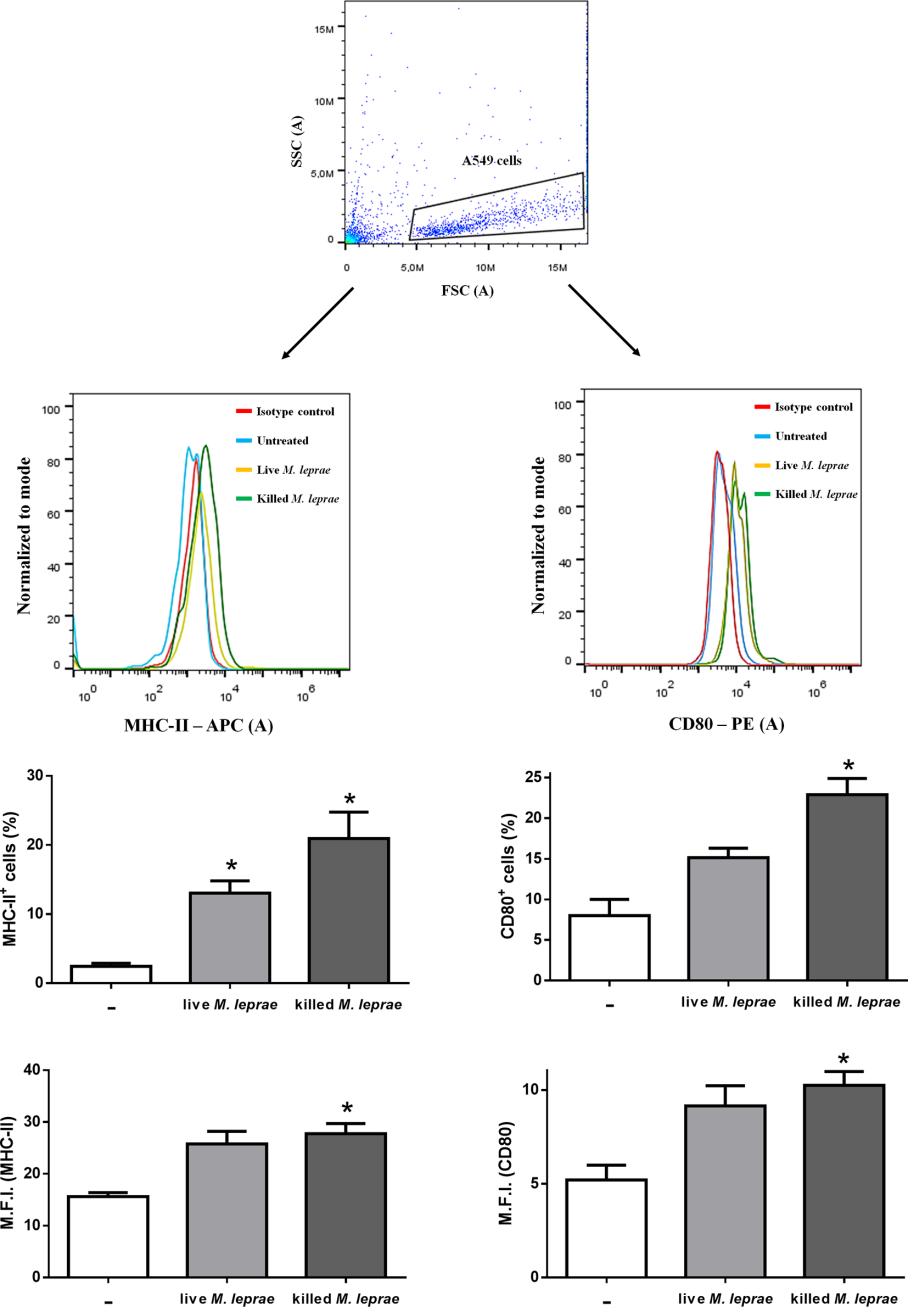
Human alveolar epithelial cells express MHC-II and CD80 in response to *M. leprae*. Flow cytometry measurement of MHC-II (left) and CD80 (right) expression on the surface of A549 cells stimulated with live or killed *M. leprae* at a bacterium:cell ratio of 50 for 48 hours. Top panel shows the gating strategy to select the A549 cells population. The representative histogram plot together with the marker’s levels of positive cells and median fluorescence intensity (M.F.I.) are shown by immunofluorescent labeling using anti-MHC-II and anti-CD80 antibodies conjugated to APC and PE, respectively. Unpaired *t*-test with differences considered statistically significant in relation to the untreated cells. *p < 0.05. Results shown as representative of 3 independent experiments performed in duplicate.

It has been shown that, in addition to MHC-II, type II pneumocytes can express several co-stimulatory molecules after different stimuli, indicating that these cells can take up, process, and then present antigens to T cells (31–36). Hence, we assessed by flow cytometry the expression of MHC-II and CD80 on the surface of A549 cells after incubation with live or killed mycobacteria (bacterium:cell ratio of 50) for 48 hours (**Figure 2**). Both the percentage of positive cells and median fluorescence intensity (M.F.I.) were significantly higher for MHC-II (**Figure 2**, left) and CD80 (**Figure 2**, right) when cells were treated with killed bacteria. Similar results were observed in cells infected with live bacteria, but statistical significance was reached only when the percentage of positive cells for MHC-II was evaluated (**Figure 2**, left). Altogether, these results indicate that independent of its viability *M. leprae* induces immune activation of epithelial cells of the respiratory tract.

### *M. leprae* Promotes Activation of the NF-κB Transcription Factor in Alveolar Epithelial Cells

**Figure 3:**
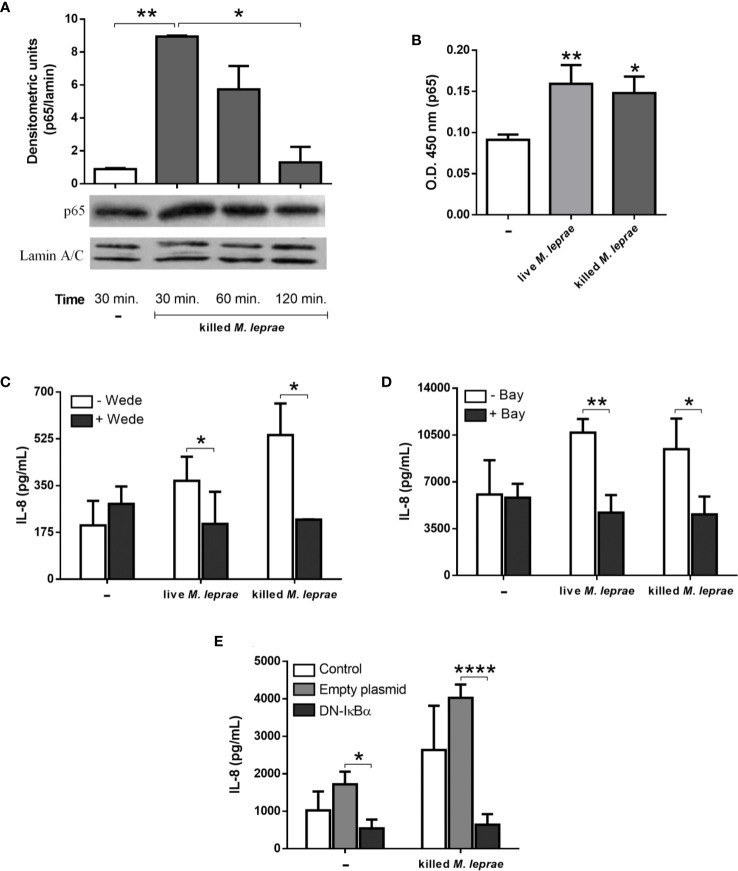
*M. leprae* induces activation of the NF-κB transcription factor in human alveolar epithelial cells. The relative levels of p65 in the nuclear extract of A549 cells were measured by immunoblotting **(A)** and by ELISA **(B)**. For immunoblotting assays, the cells were stimulated with killed *M. leprae* at a bacterium:cell ratio of 50 for 30, 60, and 120 minutes while for ELISA the cells were stimulated with live or killed *M. leprae* at bacterium:cell ratio of 50 for 30 minutes. Normalization was performed with lamin A/C and the densitometric units were arbitrary. Immunoblotting image shown as representative of 3 independent experiments performed in duplicate. **(C, D)** ELISA quantification of IL-8 levels in culture supernatants of A549 cells pretreated with wedelolactone **(C)** or Bay11-7082 **(D)** followed by stimulation with live or killed *M. leprae* at a bacterium:cell ratio of 50 for 24 hours. **(E)** ELISA quantification of IL-8 levels in culture supernatants of A549 cells transfected with DN-IκBα and stimulated with killed *M. leprae* at a bacterium:cell ratio of 50 for 24 hours. **(A–E)** Unpaired *t*-test with differences considered statistically significant between them **(A, C–E)** or in relation to the untreated cells **(B)**. *p < 0.05; **p < 0.01; ****p < 0.0001. Values ​​represent the mean ± standard deviation of at least 3 independent experiments performed in duplicate.

NF-κB is the master transcriptional factor involved in the activation of the innate immune response (37). It was then investigated if *M. leprae* activates NF-κB in A549 cells by monitoring the translocation of the p65 subunit to the nucleus. The p65 levels in the nuclear extracts were determined by immunoblotting (**Figure 3A**) and ELISA (**Figure 3B**). Cells treated with killed *M. leprae* showed significantly higher p65 subunit levels in the nucleus at 30 minutes of stimulation than in untreated cells *via* both methods, indicating activation and translocation of NF-κB in epithelial cells. Comparable results were obtained with live *M. leprae* as shown in **Figure 3B**. To determine whether NF-κB activation was linked to the increased MCP-1 and IL-8 levels observed in *M. leprae*-stimulated cells, A549 cultures were infected with live or treated with killed bacteria for 24 hours in the presence of the pharmacological inhibitors wedelolactone (**Figure 3C**) or Bay11-7082 (**Figure 3D**). Both inhibitors impair the release of NF-κB from the cytosolic complex IκB/NF-κB, avoiding NF-κB translocation to the nucleus (38–40). Treatment with wedelolactone was able to reduce the amount of IL-8 to baseline levels in cells that were infected with live or incubated with killed bacteria (**Figure 3C**). Similar results were obtained with Bay11-7082 (**Figure 3D**). Interestingly, no effect of wedelolactone on MCP-1 levels was observed (**Supplementary Figure 2**).

To confirm the involvement of NF-κB in IL-8 production in response to *M. leprae*, we also utilized DN-IκBα-transfected A549 cells, expressing a dominant-negative form of IκBα that lacks all N-terminal phosphorylation sites, thus being resistance to degradation but still with the ability of binding to NF-κB (22). Transfected cells incubated with killed *M. leprae* for 24 hours exhibited significant lower IL-8 levels in comparison with non-transfected cells or cells transfected with the empty vector used as negative control (**Figure 3E**). Identical results were obtained with transfected cells incubated for 48 hours (**Supplementary Figure 3**). Altogether, our results demonstrates that the production of IL-8 in A549 cells in response to *M. leprae* is dependent on NF-κB translocation to nucleus.

### DNA Sensing by TLR-9 Plays a Role in *M. leprae* Immune Recognition by Alveolar Epithelial Cells

TLR-9 bacterial sensing has been shown to play an important role in the immune response against mycobacteria (19, 20). Since alveolar epithelial cells express TLR-9 (41), the potential involvement of this pathway in the immune activation induced by *M. leprae* in these cells was analyzed. Firstly, the capacity of the CpG-rHlp complex to stimulate A549 cells was evaluated by measuring IL-8 secretion. For these assays, the *M. leprae* recombinant Hlp was purified from *E. coli* and some test**s** were performed for quality control. When used to stimulate PBMC, the purified recombinant protein was unable to induce detectable levels of TNF production, pointing to low levels of LPS contamination (**Supplementary Figure 4**). As expected, *M. leprae* rHlp was also found to bind DNA (**Supplementary Figure 5**). In this assay, *M. bovis* BCG genomic DNA was used since Hlp, a highly conserved protein among mycobacterial species, was shown to bind in a non-specific way to nucleic acids molecules (42, 43). Moreover, as previously shown in the context of *M. tuberculosis* Hlp, *M. leprae* rHlp, when complexed to the CpG oligo, was able to significantly increase the capacity of CpG alone to induce TNF secretion in murine macrophages (**Supplementary Figure 6B**) (17). Furthermore, by using the 30 mer synthetic peptides p2, p3 and p10 with sequences derived from different regions of Hlp (44) (**Supplementary Figure 6A**), only the p3 peptide corresponding to the DNA-binding site of the protein (43) managed to increase CpG-induced TNF secretion in macrophages (**Supplementary Figure 6B**). As expected, rHlp or the peptides alone could not induce TNF (**Supplementary Figure 6B**). Altogether, these results confirm the capacity of recombinant Hlp to enhance DNA immune recognition by TLR-9, dependent on the DNA-binding site of the protein.

**Figure 4:**
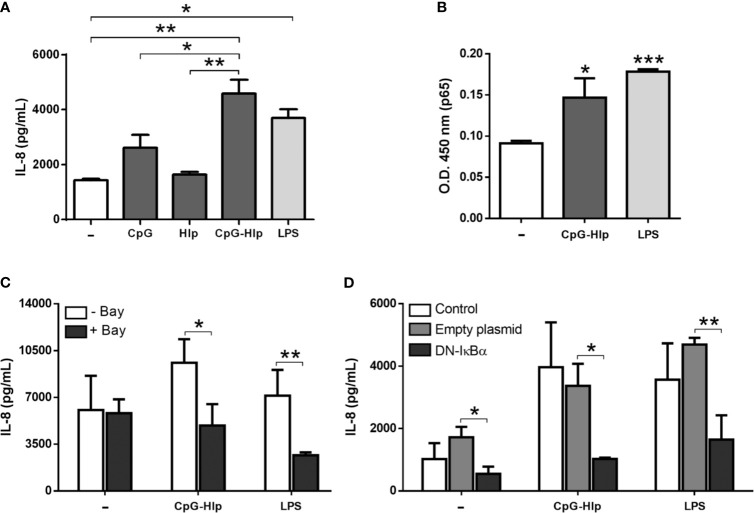
Mycobacterial CpG-Hlp complex induces immune activation of human alveolar epithelial cells. Quantification of IL-8 levels in the culture supernatant **(A, C, D)** and assessment of the relative levels of p65 subunit in the nuclear extracts **(B)** of A549 cells through ELISA. **(A)** Cells were stimulated with CpG, rHlp, or CpG-Hlp complex for 48 hours. LPS was used as a positive control. **(B)** Cells were stimulated with CpG-Hlp complex or LPS for 30 minutes. **(C, D)** Cells were pretreated with Bay11-7082 **(C)** or transfected with DN-IκBα **(D)** and then stimulated with CpG-Hlp complex or LPS for 24 hours. **(A)** ANOVA test with differences considered statistically significant between them after applying Bonferroni post-test. **(B–D)** Unpaired *t*-test with differences considered statistically significant in relation to the untreated cells **(B)** or between them **(C, D)**. *p < 0.05; **p < 0.01; ***p < 0.001. Values ​​represent the mean ± standard deviation of at least 3 independent experiments performed in duplicate.

**Figure 4A** displays the results obtained after stimulating A549 cells with the CpG-rHlp complex. CpG and rHlp alone were also used to stimulate A549 cells; and LPS was included as a positive control. Supernatants of CpG-rHlp complex-stimulated cells for 24 hours showed significantly higher levels of IL-8 when compared to those collected from cells stimulated with CpG alone while the levels of IL-8 in cultures treated with rHlp alone ended up being similar to baseline levels (**Figure 4A**). We also evaluated the capacity of CpG-rHlp complex to activate NF-κB in A549 cells. LPS, a TLR-4 ligand, was also included as control. Through ELISA we detected a significant increase of p65 in the nuclear extract after 30 minutes of stimulation with CpG-rHlp or LPS (**Figure 4B**). Next, we showed that IL-8 production in response to the CpG-rHlp is dependent on NF-κB activation (**Figures 4C, D**). A549 cells pretreated with Bay11-7082 blocked the production of IL-8 after CpG-Hlp stimulation for 24 hours (**Figure 4C**). Moreover, DN-IκBα-transfected A549 incubated with CpG-Hlp for 24 (**Figure 4D**) and 48 hours (**Supplementary Figure 7**) also showed significantly lower levels of IL-8 production when compared to control cells. Hence, the stimulation of TLR-9 by CpG-Hlp in A549 cells triggers the activation of NF-κB, which subsequently leads to the production of IL-8.

**Figure 5:**
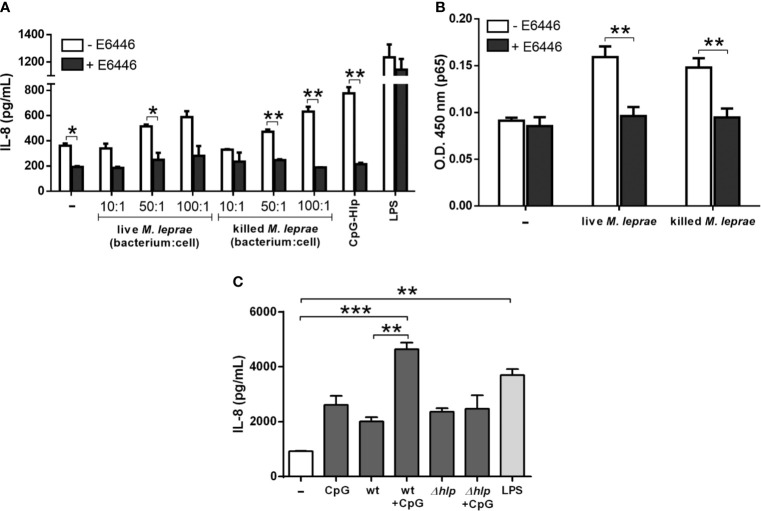
*M. leprae* induces the secretion of IL-8 in human alveolar epithelial cells *via* TLR-9/NF-κB signaling. Quantification of IL-8 levels in the culture supernatant **(A, C)** and assessment of the relative levels of p65 subunit in the nuclear extracts **(B)** of A549 cells through ELISA. **(A)** Cells were pretreated with the TLR-9 antagonist E6446 and stimulated with live or killed *M. leprae* at different bacterium:cell ratios for 24 hours. CpG-Hlp complex and LPS were used as positive and negative controls, respectively. **(B)** Cells were pretreated with E6446 and stimulated with live or killed *M. leprae* at a bacterium:cell ratio of 50 for 30 minutes. **(C)** Cells were stimulated with *M. smegmatis* wt or *Δhlp* preincubated or not with CpG at a bacterium:cell ratio of 10, or with CpG alone for 48 hours. LPS was used as a positive control. **(A, B)** Unpaired *t*-test with differences considered statistically significant between them. **(C)** ANOVA test with differences considered statistically significant between them after applying Bonferroni post-test. *p < 0.05; **p < 0.01; ***p < 0.001. Values represent the mean ± standard deviation of at least 3 independent experiments performed in duplicate.

The question as to whether TLR-9 sensing plays a role in *M. leprae* immune recognition by A549 cells was then addressed by first stimulating them with increasing bacterium:cell ratios in the presence of E6446, a synthetic antagonist of TLR-9. LPS and the CpG-Hlp complex were included as negative and positive controls, respectively. After incubation, IL-8 levels were quantified in culture supernatants. E6446 was able to completely block the secretion of IL-8 by A549 cells in response to killed *M. leprae* after 24 (**Figure 5A**) and 48 hours (**Supplementary Figure 8**) of incubation at all bacterium:cell ratios tested. Comparable results were obtained with cells infected with live *M. leprae*, although statistical significance was reached only for the bacterium:cell ratio of 50 (**Figure 5A** and **Supplementary Figure 8**). As expected, the chemokine levels in the supernatant of LPS-stimulated cells were unaffected by the presence of E6446. In contrast, a complete inhibition of IL-8 secretion was observed in response to the CpG-Hlp complex. E6446 was also able to block the nuclear translocation of p65 induced by live/killed *M. leprae* in A549 cells as shown in **Figure 5B**. Altogether, these results demonstrate that production of IL-8 in response to *M. leprae* infection in A549 cells occurs *via* TLR-9/NF-κB.

### Surface-Exposed Hlp Binds Extracellular DNA, Augmenting Mycobacterial Immunostimulatory Capacity

Since TLR-9 seems to play a prominent role in *M. leprae* immune recognition by alveolar epithelial cells, the hypothesis was raised that bacterial extracellular DNA anchored to Hlp molecules present on the bacterial surface could act as potent TLR-9 ligands by specifically triggering this immune activation pathway during infection. To test this hypothesis, experiments were conducted using *M. smegmatis* wild type (wt) and *Δhlp* mutant strains. This mycobacterium was chosen due to the complete inability to generate mutants in *M. leprae*. In addition, the Hlp protein is a highly conserved protein among the different species of mycobacteria (45). A549 cells were treated with *M. smegmatis* wt and *Δhlp* strains previously incubated or not with CpG oligo; and, after 48 hours, IL-8 levels were measured in culture supernatants. LPS-incubated A549 cells were included as positive controls. Both *M. smegmatis* strains were able to induce IL-8 production in A549 cells at similar levels. However, while pre-incubation of the wt strain with CpG resulted in approximately 2.3-fold more IL-8 when compared to cells stimulated with mycobacteria alone, this increment was not observed in the *Δhlp* strain (**Figure 5C**). These results strongly suggest that surface-exposed Hlp interacting with bacterial extracellular DNA forms DNA-Hlp complexes that may act as potent TLR-9 ligands during their interaction with host cells.

## Discussion

One of the most important gaps in knowledge concerning the natural course of *M. leprae* infection in humans revolves around the earliest interactions between the pathogen and the respiratory tract, considered the main infection route of the bacillus. Earlier studies have shown that, after airborne exposure or intranasal infection of mice, *M. leprae* can reach the lungs and is able to invade and survive inside pulmonary epithelial cells (11, 12). Given the proven weight of the respiratory pulmonary epithelium in generating an immune response against invading microorganisms (7), the present study investigated immune pathways activated *in vitro* in *M. leprae-*challenged alveolar epithelial cells.

In most experiments performed, the effects of live and killed *M. leprae* were compared, since in other cell types modulation of several host cell functions by *M. leprae* was shown to be induced only by live bacterium (46–48). To our surprise, in the case of A549 cells, all effects herein demonstrated were proven to be independent of bacterial viability. Initially, the capacity of *M. leprae* to induce the secretion of pro-inflammatory mediators in A549 cells was analyzed. Among the mediators evaluated, the chemokines MCP-1 and IL-8 showed higher levels in the supernatants of *M. leprae*-stimulated cells. *M. leprae* also augmented the secretion of IL-8 in human primary nasal epithelial cells, reinforcing that this response might occur in *M. leprae-*exposed individuals during bacterial entry through the respiratory airways.

MCP-1 is a chemoattracting molecule of CD4^+^ T cells and monocytes (49) whereas IL-8 predominantly attracts neutrophils, which are the first inflammatory cells to migrate to the infection site, restricting bacterial spread. The production of both chemokines by alveolar epithelial cells subsequent to *M. tuberculosis* infection has already been well demonstrated (50–55). The protective role of MCP-1 in mycobacterial infection has been documented in several studies as well. The presence of circulating MCP-1 serum levels was shown to be significantly greater in pulmonary as compared to extra-pulmonary tuberculosis patients and endemic individuals (56). Moreover, after being infected with BCG, mice overexpressing MCP-1 in type-II alveolar epithelial cells demonstrated increased pools of lung mononuclear phagocytes and significantly decreased mycobacterial loads in the bronchoalveolar space together with the rapid resolution of lung granuloma formation (57). Regarding IL-8, bronchoalveolar fluids from tuberculosis patients showed a dramatic uptick in the number of neutrophils in correlation with elevated concentrations of the chemokine (58, 59). Some studies also described the capacity of *M. tuberculosis*-induced IL-8 to act as a chemoattractant of CD4^+^ and CD8^+^ T cells in pulmonary granuloma, modulating the adaptive immune response (49, 60, 61).

Defensins are cationic peptides that participate in innate immunity due to their microbicidal activities, mainly through bacterial membrane permeabilization (62). Indeed, several studies indicate that A549 cells express hβD-2 after infection by mycobacteria, leading to a decrease in the intracellular bacillary load due to bacterial killing (27–30). In an earlier study, we showed that *M. leprae* was able to stay alive for at least 10 days within A549 cells (12). However, in that work cells were infected with 10x lower the bacterium:cell ratio needed to induce the expression of hβD-2, according to our current data (bacterium:cell ratio of 100). Thus, future studies are needed to confirm that hβD-2 produced by epithelial cells in response to *M. leprae* infection can promote bacterial killing.

In addition, significant hβD-2 levels have been detected in bronchoalveolar lavage fluid from patients with *M. avium-intracellulare* infection (63), corroborating the involvement of such peptides in the host defense and local remodeling of the respiratory tract in mycobacterial infection. Interestingly, one *in vitro* study showed low hβD-2 production in alveolar macrophages infected with *M. tuberculosis*, unlike the alveolar epithelial cells that produced large amounts of these peptides under the same conditions (27), inferring that epithelial cells, and not macrophages, are the main sources of hβD-2 during a primary mycobacterial lung infection. Defensins also exhibit chemotactic properties in initiating and regulating the immune response (64, 65). As to hβD-2 specifically, it is identified as capable of primarily attracting T cells but dendritic and mast cells as well (66–68). In a mouse model of pulmonary tuberculosis, β-defensin-2 immunostaining was detected in cells with dendritic morphology located nearby mediastinal lymph nodes, indicating its contribution to the establishment of a Th1 response, thus bridging the innate and adaptive immune responses (69).

In a next step, the effect of *M. leprae* stimulation of A549 cells on the expression of MHC-II and the co-stimulatory molecule CD80 was examined. Our data indicate that *M. leprae* was able to induce MHC-II and CD80 expression. These results suggest that, during post-*M. leprae* infection, alveolar epithelial cells not only recruit T cells as a consequence of producing pro-inflammatory mediators, but also assist in T cell activation by presenting antigens.

For the purpose of evaluating the mechanism of *M. leprae*-induced immune activation in alveolar epithelial cells, the involvement of NF-κB transcription factor was ascertained. The bacillus was found to be capable of inducing NF-κB activation in A549 cells by promoting the translocation of the p65 subunit to the nucleus. Moreover, cells treated with wedelolactone or Bay11-7082, two NF-κB inhibitors, were unable to produce IL-8 in response to *M. leprae*. Additionally, A549 cells overexpressing a dominat-negative IκBα mutant displayed lower levels of IL-8 in response to *M. leprae*. Interesting, in contrast to the clear activation of NF-κB in alveolar epithelial cells herein demonstrated, *M. leprae* has been shown to be both a weaker stimulator and even an inhibitor of this transcriptional factor in monocytes and Schwann cells (70, 71).

No effect on MCP-1 production was observed when supernatant MCP-1 was measured in A549 cells treated with wedelolactone, suggesting that the regulation of this chemokine is governed by a distinct mechanism. In fact, several studies have shown that different transcriptional pathways, i.e., other than NF-κB, are involved in MCP-1 production (72–74). In the context of lung epithelial cells, the IL-1β-induced MCP-1 expression in murine alveolar type II epithelial cells required multiple transcriptional factors in addition to NF-κB; namely, c-Jun N-terminal kinase (JNK), CCAAT/enhancer-binding proteins (C/EBPβ and C/EBPδ) and specificity protein 1 (Sp1) (75).

One of the pathways leading to NF-κB activation is the recognition of PAMPs by TLRs. Of interest, TLR-9 is one of the intracellular TLRs expressed by human alveolar epithelial cells (41) that has been considered in the context of mycobacterial infection due to its capacity to sense microbial DNA and mediate protective responses against infection (19, 21). Meeting expectations, A549 cells secreted IL-8 in response to the CpG-Hlp complex, a known mycobacterial TLR-9 ligand. Moreover, in agreement with previous reports (17, 21), the combination of rHlp with CpG almost doubled the levels of secreted IL-8 when compared to cells stimulated with CpG alone. One possible explanation for the synergistic effect of Hlp on CpG may be due to the ability of the protein to bind simultaneously to components of the extracellular matrix (14, 15, 45, 76) and to DNA (42, 43). The binding of Hlp to extracellular matrix components would promote the endocytosis of the CpG-Hlp complex, facilitating its subsequent recognition by the TLR-9 in the endosomal compartments. Moreover, histones are also able to induce DNA curvature, an effect that appears to significantly increase DNA binding by TLR-9 (77).

Based on this information, the next analysis centered on the potential involvement of TLR-9 activation in the *M. leprae*-induced immune activation in these cells. For this purpose, we employed E6446, a very selective antagonist for TLR-9 used in several previous reports (21, 78–82). The inclusion of TLR-4 and TLR-9 ligands as negative and positive controls, respectively, in our assays reinforced the specificity of E6446 towards TLR-9. A549 cells pre-treated with E6446 completely blocked the secretion of *M. leprae*-induced IL-8, elucidating that, perhaps, once internalized, bacterial DNA sensing by TLR-9 constitutes an important innate immune pathway becoming activated in infected respiratory epithelial cells. Moreover, by blocking TLR-9, the translocation of p65 to the nucleus and IL-8 production were inhibited, linking TLR-9 signaling with NF-κB activation and IL-8 induction in A549 cells stimulated with *M. leprae*.

Studies in a murine model of experimental malaria have reinforced the immunostimulatory properties of DNA-protein complexes (83, 84). The DNA-histone complex proved to be the main component in the plasmodium capable of activating dendritic cells and inducing the production of inflammatory mediators. The above-mentioned authors also demonstrated that histones facilitate the internalization of the DNA molecule and its subsequent recognition by TLR-9. These and several other studies have strengthened the idea that (naked) DNA *per se* might likely be an immunologically weak molecule, but its association with such DNA-binding proteins as histones, could transform it into a molecule with stronger immunostimulatory properties (17, 21, 85).

Previous studies have found the presence of DNA on the surface of *M. tuberculosis* (86, 87), possibly as a result of bacterial lysis or active secretion, as has already been seen in *M. avium* (88, 89). A prior study showed that *M. avium* cultivated in a “phagosome-mimicking” model secreted a high number of DNA-containing membrane vesicles (89). These same authors were also able to determine, by way of freeze-fracture transmission electron microscopy, the presence of these vesicles inside the macrophages as well as the presence of extracellular DNA in the matrix of *M. avium* biofilms (88). It is well established that the Hlp protein is present on the surface of *M. leprae* (12, 16, 25). So, it is reasonable to speculate that extracellular DNA anchored to Hlp constitute a major TLR-9 ligand triggering this immune activation pathway during mycobacterial infection. This hypothesis was reinforced when a mutant *M. smegmatis* strain for the *hlp* gene was employed. Pretreatment of the *M. smegmatis* wt strain with CpG increased the secretion of IL-8 by A549 cells, which did not occur with regard to the *Δhlp* strain. These assays made it possible to conclude that Hlp promotes the binding of DNA to the bacterial surface, leading to the subsequent activation of TLR-9 upon bacterial internalization.

**Figure 6:**
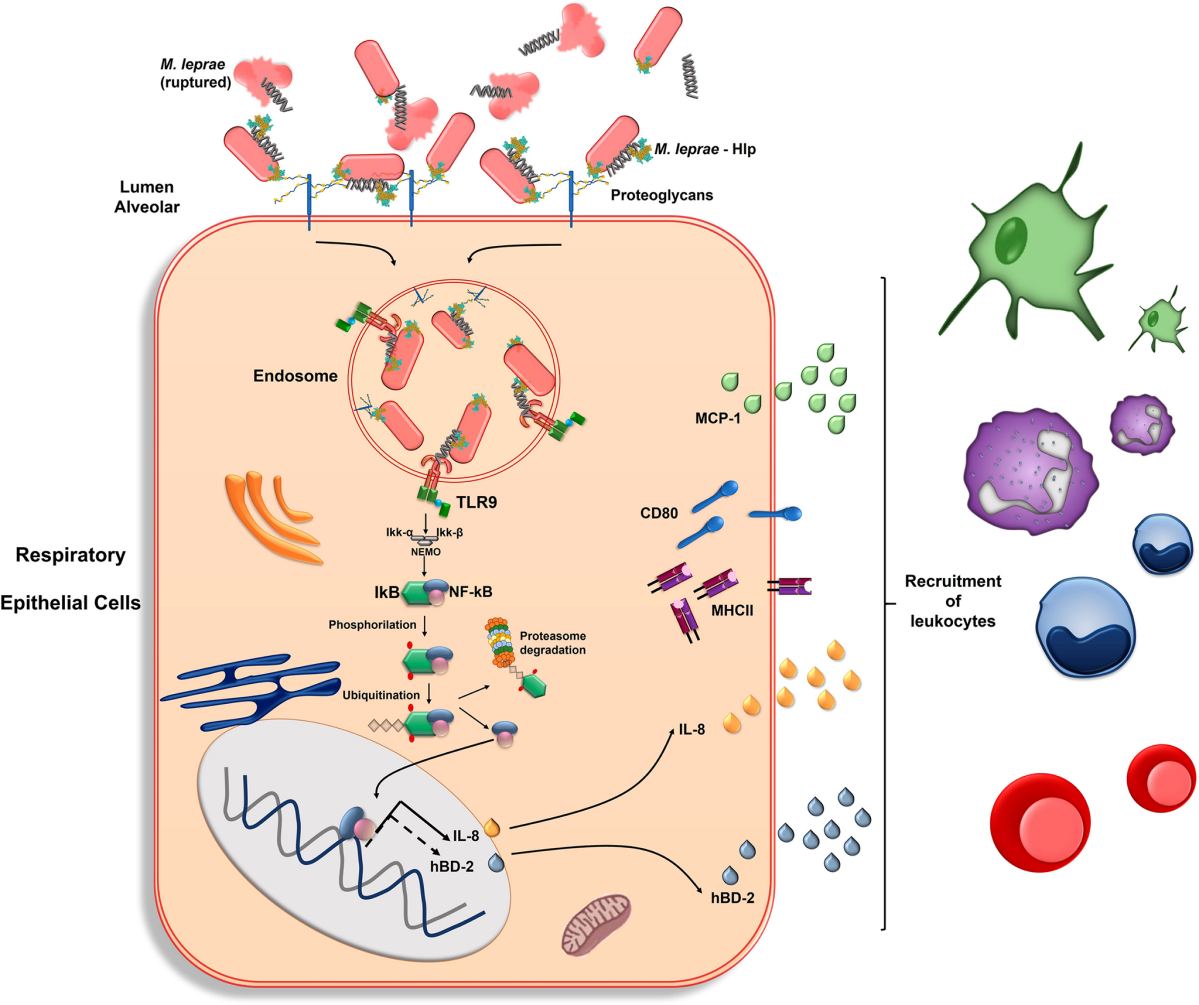
Representative model of *M. leprae-*respiratory epithelial cell interaction with emphasis on the role of bacterial DNA-Hlp complex recognition in cellular immune activation. DNA molecules, probably derived from the leakage of dead bacteria, bind to surface-exposed Hlp, allowing the deposition and accumulation of DNA-Hlp complexes on the surface of viable and dead bacilli. Hlp mediates the adhesion and internalization of *M. leprae* to respiratory epithelial cells *via* its (Hlp) capacity to bind extracellular matrix proteoglycans. In the endosome, the DNA-Hlp complex interacts with the TLR-9, leading to activation and nuclear translocation of the NF-κB transcription factor with subsequent induction of IL-8. The hβD-2 is also induced, possibly *via* NF-κB (90–92) (dotted arrow). MCP-1 is also produced in response to *M. leprae*, however, in a NF-κB-independent manner. *M. leprae* also induces the expression of MHC-II and co-stimulatory molecules like CD80. This immune activation mediates the recruitment of leukocytes to the infection site, represented by dendritic cells (green), neutrophils (lilac), monocytes (blue) and T cells (red), contributing to the generation of a protective adaptive immune response.

**Figure 6** summarizes our current model of *M. leprae*-airway epithelial cell interaction and the immune activation pathways triggered upon infection. DNA molecules, probably derived from the leakage of dead bacteria, bind to surface-exposed Hlp, allowing for the deposition and accumulation of DNA-Hlp complexes on the surface of both viable and dead bacilli. According to our **Supplementary Data** and previous findings (43), DNA binding to Hlp occurs *via* the prokaryotic DNA-binding motif located at the N-terminal half of the protein. Hlp mediates the adhesion and internalization of *M. leprae* to respiratory epithelial cells *via* its (Hlp) capacity to bind extracellular matrix proteoglycans such as heparan sulfate present on the cell surface. Once the bacilli reach the endosomal compartment, the DNA-Hlp complexes are recognized by the TLR-9 receptor, triggering a network of signals leading to NF-κB activation and the production of IL-8. *M. leprae* infection also induces MCP-1 and antimicrobial peptide production as well as the expression of MHC-II and co-stimulatory molecules like CD80. The signaling pathways triggered by *M. leprae* to induce these activation markers were not investigated in the present study, but bacterial recognition by other PRRs might play a role and should be explored in future studies. The *M. leprae*-induced immune activation of airway epithelial cells will mediate the recruitment of leukocytes to the infection site, contributing to the generation of a protective adaptive immune response.

Most individuals exposed to *M. leprae* are able to build a protective Th1-based immune response against the infection and, consequently, do not progress to active disease (6). Therefore, based on the data herein demonstrated it is reasonable to speculated that the ability of *M. leprae* to immune activate respiratory epithelial cells represents a very early event during infection that may be crucial to the development of this protective response. Indeed, mucosal vaccines are able to induce both local and systemic immunity against infectious agents (93–95). Of interest, the addition (or co-delivery) of potent adjuvants such as TLR-9 agonists has been described as efficient in potentiating a mucosal vaccination against mycobacterial infections (96).

In a scenario bereft of a leprosy vaccine, the findings described above may shed some much-needed light on creating potentially effective strategies toward achieving a viable vaccine in the near term. Moreover, since Hlp is a very conserved protein, we foresee its potential use when combined with DNA, both as a good immunogen as well as an adjuvant in immune interventions such as in intranasal vaccination against mycobacteria.

## Data Availability Statement

The original contributions presented in the study are included in the article/**Supplementary Material**. Further inquiries can be directed to the corresponding author.

## Ethics Statement

The studies involving human participants were reviewed and approved by Pedro Ernesto University Hospital, the State University of Rio de Janeiro (CEP 2306/HUPE) and the Oswaldo Cruz Foundation (FIOCRUZ) Ethical Committee (CEP 483/08). The patients/participants provided their written informed consent to participate in this study.

## Author Contributions

AD, CMS, COS, and MP conceived and designed the experiments. AD, CMS, COS, NL, JS, AV, and PR performed the experiments. AD, CMS, COS, AC, MM, UL, MB-P, and MP analyzed the data. AD, CMS, and MP wrote the paper. All authors contributed to the article and approved the submitted version.

## Funding

This work was supported by grants awarded to MCVP by the Conselho Nacional de Desenvolvimento Científico e Tecnológico (CNPq; 310155/2017-7), and the Fundação Carlos Chagas Filho de Amparo à Pesquisa do Estado do Rio de Janeiro (FAPERJ; 110.527/2014). AD was the recipient of a fellowship from Oswaldo Cruz Institute (IOC/FIOCRUZ/Brazil). The funders had no role in study design, data collection and analysis, decision to publish, or preparation of the manuscript.

## Conflict of Interest

The authors declare that the research was conducted in the absence of any commercial or financial relationships that could be construed as a potential conflict of interest.

## Publisher’s Note

All claims expressed in this article are solely those of the authors and do not necessarily represent those of their affiliated organizations, or those of the publisher, the editors and the reviewers. Any product that may be evaluated in this article, or claim that may be made by its manufacturer, is not guaranteed or endorsed by the publisher.
