# Supplementary material for: TLR-9 Plays a Role in Mycobacterium leprae-Induced Innate Immune Activation of A549 Alveolar Epithelial Cells
Source: Front Immunol. 2021 Aug 12;12:657449. doi: 10.3389/fimmu.2021.657449 (PMC8397448; doi:10.3389/fimmu.2021.657449)
Supplement: Supplementary file 1 [file DataSheet_1.doc]

**Supplemental Figure 1: Secretion of chemokines in response to *M. leprae* in human alveolar epithelial cells.** ELISA quantification of MCP-1 **(A)** and IL-8 **(B)** levels in the culture supernatant**s** of A549 cell stimulated with live or killed *M. leprae* at different bacterium:cell ratios for 48 hours. **(A)** Unpaired *t*-test with differences considered statistically significant in relation to the untreated cells. **(B)** ANOVA test with differences considered statistically significant in relation to the untreated cells comparing the different doses by applying a linear-trend post-test. ** p <0.01; *** p <0.001. Values ​​represent the mean ± standard deviation of at least 3 independent experiments performed in duplicate.

**Supplemental Figure 2: Secretion of MCP-1 induced by *M. leprae* in human alveolar epithelial cells is independent of activation of the NF-κB transcription factor.** ELISA quantification of MCP-1 levels in the culture supernatants of A549 cells pretreated with wedelolactone and stimulated with live or killed *M. leprae* at a bacterium:cell ratio of 50 for 24 hours. Values ​​represent the mean ± standard deviation of 3 independent experiments performed in duplicate.

**Supplemental Figure 3: Secretion of IL-8 induced by *M. leprae* in human alveolar epithelial cells is dependent on NF-κB activation.** ELISA quantification of IL-8 levels in the culture supernatants of A549 cells transfected with DN-IκBα and stimulated with killed *M. leprae* at a bacterium:cell ratio of 50 for 48 hours. Unpaired *t*-test with differences considered statistically significant between them. *** p <0.001. Values ​​represent the mean ± standard deviation of 3 independent experiments performed in duplicate.

**Supplemental Figure 4: Secretion of TNF by PBMCs in response to *M. leprae* rHlp.** ELISA quantification of TNF levels in the PBMCs supernatants obtained from healthy individuals and cultured in the presence of *M. leprae* rHlp for 24 hours. *E. coli* LPS was used as a positive control. Values ​​represent the mean ± standard deviation of 2 independent experiments performed in duplicate.

**Supplemental Figure 5: Binding of the *M. leprae* rHlp protein to mycobacterial genomic DNA.** Polystyrene microplate wells coated with DNA were incubated with increasing concentrations of rHlp. The wells were then incubated with the anti-Hlp monoclonal antibody 5G9 and, lastly, with a rabbit anti-mouse IgG peroxidase conjugate. Peroxidase activity was revealed via hydrogen peroxide and tetramethylbenzidine (TMB). The result was monitored by absorbance analysis at 450 nm. Result shown as representative of 3 independent experiments performed in duplicate.

A)

**Supplemental Figure 6: Secretion of TNF by RAW 264.7 macrophages in response to CpG combined with *M. leprae* rHlp or derived peptides. (A)** *M. leprae* Hlp amino acid sequence. The DNA-binding site is highlighted in yellow and the amino acids in red make up the extracellular matrix binding regions. The sequences corresponding to the p2, p3 and p10 synthetic peptides are indicated. **(B)** ELISA quantification of TNF levels in the supernatant of RAW 264.7 macrophagic cell cultures stimulated with CpG alone or in combination with rHlp, p2, p3, or p10 for 24 hours. LPS was used as a positive control. ANOVA test with statistically significant differences between them after applying Bonferroni post-test. * p <0.05; ** p <0.01; *** p <0.001. Values ​​represent the mean ± standard deviation of 3 independent experiments performed in duplicate.

**Supplemental Figure 7: Secretion of IL-8 induced by CpG-Hlp in human alveolar epithelial cells is dependent on NF-κB activation.** ELISA quantification of IL-8 levels in the culture supernatants of A549 cells transfected with DN-IκBα and stimulated with CpG-Hlp complex for 48 hours. LPS was used as control. Unpaired *t*-test with differences considered statistically significant between them. * p <0.05; ** p <0.01; *** p <0.001. Values ​​represent the mean ± standard deviation of 3 independent experiments performed in duplicate.

**Supplemental Figure 8: Secretion of IL-8 induced by *M. leprae* in human alveolar epithelial cells via TLR-9 recognition.** ELISA quantification of IL-8 levels in the culture supernatants of A549 cells pretreated with the TLR-9 antagonist (E6446) and stimulated with live or killed *M. leprae* at different bacterium:cell ratios for 48 hours. LPS and the CpG-Hlp complex were used as controls. Unpaired *t*-test with differences considered statistically significant between them. * p <0.05; ** p <0.01. Values ​​represent the mean ± standard deviation of 3 independent experiments performed in duplicate.
